# Nonlinear effects and effect modification at the participant-level in IPD meta-analysis part 2: methodological guidance is available

**DOI:** 10.1016/j.jclinepi.2023.04.014

**Published:** 2023-05-03

**Authors:** Nadine Marlin, Peter J. Godolphin, Richard L. Hooper, Richard D. Riley, Ewelina Rogozińska

**Affiliations:** a Methodology Research Unit, Centre for Evaluation and Methods, Wolfson Institute of Population Health, Queen Mary University of London, 58 Turner Street, London E1 2AB, UK; b MRC Clinical Trials Unit at University College London, Institute of Clinical Trials and Methodology, 90 High Holborn, London WC1V 6LJ, UK; c Institute of Applied Health Research, College of Medical and Dental Sciences, University of Birmingham, Birmingham B15 2TT, UK

**Keywords:** Individual participant data meta-analysis, Methodology, Effect modification, Interaction, Nonlinear, Sample size, Fractional polynomials, Restricted cubic splines, One- and two-stage models

## Abstract

**Objectives:**

To review methodological guidance for nonlinear covariate-outcome associations (NL), and linear effect modification and nonlinear effect modification (LEM and NLEM) at the participant level in individual participant data meta-analyses (IPDMAs) and their power requirements.

**Study Design and Setting:**

We searched Medline, Embase, Web of Science, Scopus, PsycINFO and the Cochrane Library to identify methodology publications on IPDMA of LEM, NL or NLEM (PROSPERO CRD42019126768).

**Results:**

Through screening 6,466 records we identified 54 potential articles of which 23 full texts were relevant. Nine further relevant publications were published before or after the literature search and were added. Of these 32 references, 21 articles considered LEM, 6 articles NL or NLEM and 6 articles described sample size calculations. A book described all four. Sample size may be calculated through simulation or closed form. Assessments of LEM or NLEM at the participant level need to be based on within-trial information alone. Nonlinearity (NL or NLEM) can be modeled using polynomials or splines to avoid categorization.

**Conclusion:**

Detailed methodological guidance on IPDMA of effect modification at participant-level is available. However, methodology papers for sample size and nonlinearity are rarer and may not cover all scenarios. On these aspects, further guidance is needed.

## 1 Introduction

Personalised medicine, also termed precision medicine, is becoming increasingly relevant in health care decision-making. It requires understanding of how treatment effects may vary depending on individual characteristics, for example, gender or age. Individual participant data meta-analysis (IPDMA) of randomized trials (RCTs) are often well suited to investigate such complex participant-level re-lationships, due to increased sample size over single trials and greater methodological flexibility compared to meta-analysis based on aggregated data [1–3]. This flexibility enables a reliable assessment of linear effect modification (LEM), nonlinear covariate-outcome associations (NL), and nonlinear effect modification (NLEM). Terminology varies in the literature [4] (Box 1).

IPDMA of such complex relationships can provide a more nuanced understanding of which patients benefit most from interventions, thereby optimising how treatments are used in practice [5]. For example, Leijten et al. showed that children with more severe conduct problems gained the most from the Incredible Years program [6]. Additionally, such analyses may also identify a need for more effective interventions in certain subgroups, for example, in pulmonary arterial hypertension patients [7]. Interpretation can be challenging, and appropriate expertise is required to properly interpret and communicate such complex analyses.

Effect modification should be considered during the design stage of IPDMAs; however this rarely occurs [8]. Planning a sufficiently powered treatment effect modification analysis requires considerably larger sample sizes than for the overall treatment effect [9,10]. Although researchers have limited impact on sample size (acquired IPD), power considerations have many benefits, such as indicating whether planned analyses have the potential to provide meaningful results. They may support decisions on which analysis to plan or even which trials to focus on for data retrieval [11].

Analysis methods for effect modification should separate within- and across-trial information, to avoid the potential for aggregation bias impacting participant-level relationships. This occurs when a between-trial relationship (e.g., trials that include a higher proportion of women find larger treatment effects) is misinterpreted as a within-trial relationship (the treatment effect is larger in women compared to men) [12–15].

Our previous review found few IPDMA studies reporting power considerations for analysis of effect modification and often inadequate methodology and reporting of LEM, NL and NLEM analyses [16]. It is unclear what guidance for these complex analyses is available.

In this article, we present findings of a review of current methodology for examining LEM, NL and/or NLEM at the participant-level in IPDMA, and summarize recommendations. This overview will serve anyone involved in the planning, analysis, or review of an IPDMA in exploring the range of potential approaches for their specific IPDMA project.

## 2 Methods

### 2.1 Literature review

The detailed methods including search strategy are described elsewhere [16]. In brief, we searched six databases from 01 January 2015, to 04 November 2020, without language restrictions for methods papers describing approaches for IPDMA of LEM, NL, or NLEM. The search strategy was developed in discussion with an information specialist and was sensitive and comprehensive, therefore suitable to identify research studies and methodology papers.

The search was guided by a prospectively registered protocol (CRD42019126768) and recommendations on the conduct of methodological studies [17]. Reference lists and citation indices of relevant publications were hand searched for further relevant methodological papers up to 01 November 2022. Due to the low number of publications on power calculations for LEM, NL, or NLEM in IPDMA we also included references on this topic published before 01 January 2015.

### 2.2 Eligibility criteria

Methodology publications were eligible if they described, reviewed, assessed, or compared methodology for IPDMA of RCTs addressing effect modification, subgroup effects, NL and/or power calculations. We excluded methodology articles on network meta-analysis, nonfrequentist methods and those dealing with summary-level data only or where the full text was not accessible.

### 2.3 Screening

One researcher (NM) identified potentially relevant IPD-MA methods papers by screening titles and abstracts. All potentially relevant IPDMA methodology papers underwent full-text review by one researcher (NM). If uncertain, the articles were discussed with other experienced members of the team (RR, PG).

### 2.4 Data extraction

Data were extracted using a prospectively developed excel spreadsheet by one researcher (NM). In addition, a narrative synthesis was developed by NM and discussed within the team.

We extracted general information, the analysis methods considered, the approach, and if available, aims, recommendations, and limitations (Box 2).

## 3 Results

**Fig. 1:**
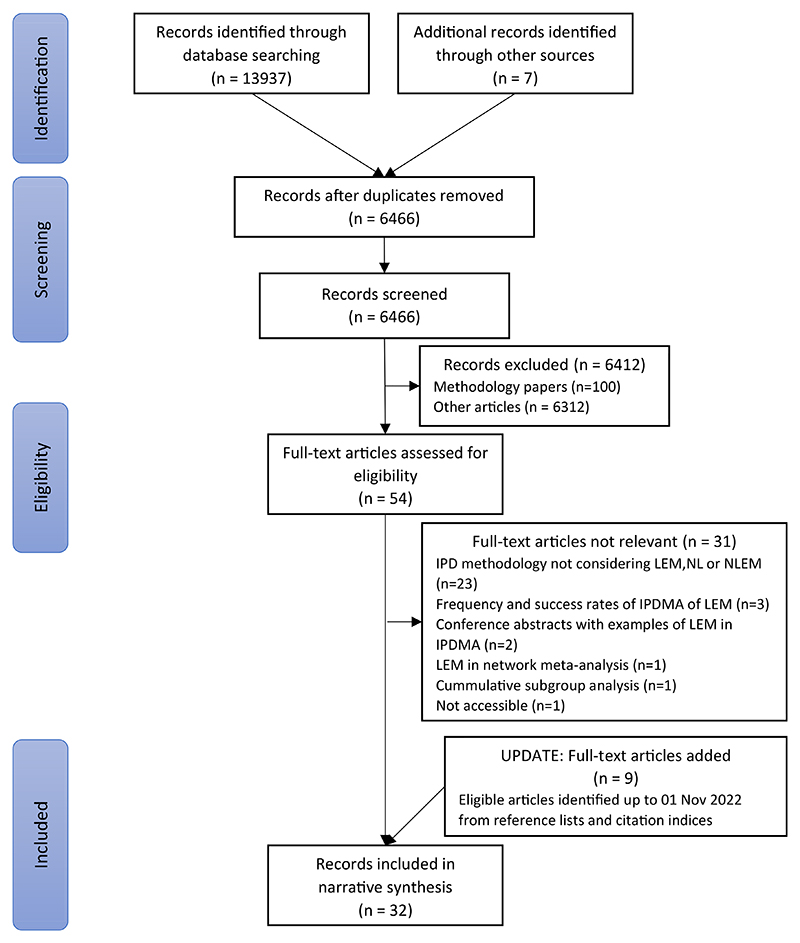
Flow diagram.

Database searches identified 6,466 unique records including 54 potentially eligible methodology articles published between 2015 and 2020 (Fig. 1). They were considered in full text. Of these, 23 were relevant and included in the narrative synthesis together with a further seven articles published after November 2020 and identified up to 01 November 2022, and two articles published before January 2015. These 32 relevant articles mainly focused on the analysis of subgroup effects and effect modification and are considered below. References of excluded articles are listed in the Appendix.

### 3.1 IPDMA approaches for subgroup effects and linear effect modification

Table 1 presents 21 methodological papers and 1 book chapter considering subgroup effects and effect modification at the participant-level published since 2015. Many of the methodologies presented draw on work by earlier authors, of which most are referenced in the reviews by Riley, Fisher, Gao, Hua, and Simmonds [2,13,18,19,33,35].

#### 3.1.1 Comparison of one- vs. two-stage and common vs. random effects models for subgroup effects and interaction terms

IPDMA of effect modification can either be performed in two stages, where analyses are performed within each trial and the summary measures combined, or in one stage, where individual level data from all trials is analyzed together while accounting for clustering by trial [23] (Box 1).

Riley and colleagues provide comprehensive guidelines on analysis of effect modification in one- or two-stage settings [2,19]. Both publications highlight the problems with categorization of continuous covariates, the challenges of one-stage approaches when it comes to separating within- and across trial variation and the need to power IPDMAs for analysis of subgroup effects.

We identified three articles comparing common-effect and random-effects and one-stage and two-stage models through simulation [14,30,31]. Belias and Kontopantelis advocate a one-stage approach although Kontopantelis’ simulation studies merged across and within-trial relationships, and are therefore prone to aggregation bias. Morris warned that one-stage models are far easier to specify incorrectly but found little difference between two- and one-stage approaches if the models are correctly specified. This is in line with the theoretical comparison performed by Burke [23]. Two further articles by da Costa and Hua compared one-stage approaches with both emphasizing the need to separate within- and across-trial variation [27,33]. Walker used an IPD dataset to compare two- and one-stage approaches with varying assumptions of random effects and found the effect modification estimates to be similar [32]. Convergence and stability issues may dictate the choice of method and prespecification of methods is vital to avoid data dredging.

#### 3.1.2 Other approaches to effect modification

The articles described above consider subgroup analysis or inclusion of prespecified interaction terms into the meta-analysis model. The following four articles describe alternatives when dealing with effect modification.

Vo suggests separation of “case-mix heterogeneity” (i.e., effect modifiers) and “beyond case-mix heterogeneity” (i.e., other differences between studies such as design) [21]. In the presence of heterogeneity an overall treatment effect can still be clinically relevant if it is standardised to a reference population of interest.

Two articles by Fokkema and Mistry describe the exploration of larger numbers of potential subgroup effects using tree-based methods [22,29]. Amalgamation of within- and across-trial variation is not addressed in these articles. Jiao presents a mapping approach for investigating multiple covariates across datasets using two-step approach that first links study-specific vectors of parameters and then estimates hyperparameters using a multivariate random-effects meta-analysis model [20].

The META-STEPP approach estimates subpopulation treatment effects based on overlapping patient subpopulations [34]. Treatment effects are analyzed by standard common-effects meta-analysis methodology. This approach may be useful for larger numbers of effect modifiers and complex effect modification but does not separate within- and across trial variation.

Four further papers address specialized issues when analyzing effect modification: use of pseudo IPD [26], analysis of repeated measures data [28], measures of heterogeneity [1] and multivariate meta-analysis of multiple outcomes [25].

#### 3.1.3 Reporting

Fisher reviewed the methods used to analyse effect modification in IPDMA research studies published between 2011 and 2014 [13]. Of those few with sufficient description, most did not separate within- and across-trial variation correctly and were at risk of aggregation bias. Two-stage IPDMAs of interaction terms inherently address this issue, whilst one-stage approaches require more care in model specification. A review of cancer IPD studies by Gao found a similar lack in clear reporting and appropriate analysis methods used, with all IPDMAs that included continuous covariates categorizing them when assessing effect modification [18].

Schandelmaier developed the ICEMAN tool to score the credibility of effect modification analyses [4]. Credibility is gained on factors including the use of random-effects models, the separation of within- and between-study effects and avoiding categorizing continuous covariates.

#### 3.1.4 Statistical software

Fisher published the Stata command (IPDMETAN), which performs both stages of a two-stage IPD meta-analysis [24]. Effect modification analysis and inclusion of nonlinear terms is possible.

### 3.2 IPDMA approaches for nonlinear covariate-outcome relationships and nonlinear effect modification

We found no published reviews of IPDMA methods for NL. We identified six methodological papers and one book chapter that described methods for either NLEM or nonlinear relationships between covariates and outcomes (Table 2).

Splines and fractional polynomials can be used to model nonlinear covariate-outcome relationships and effect modification in two-stage models [2,37–39]. Best fitting nonlinear effect (modification) is identified in the first stage and then combined in the second stage pointwise (meta-curve [41]) or using multivariate meta-analysis (mvmeta [42]). The former allows for study-specific polynomial functions, the latter only for common functions. White also show the advantages of allowing for nonlinear covariate-outcome relationships over the commonly used categorization approach [39].

Riley and colleagues suggest using restricted cubic splines for their increased flexibility compared to fractional polynomials and combining them using multivariate meta-analysis [2,19]. If a one-stage approach is desired this can be done by stratifying the trial parameters outside the interaction term. They highlight that effect modification may depend on the scale of the analysis and refer to a theoretical example by Shrier and Pang who found a statistically significant interaction when analyzing odds ratios but not when analyzing risk ratios [43]. This is due to differences in baseline risk, and can therefore also be seen, for example, in survival analysis of time-to-event outcomes.

Belias compares four types of splines and three pooling methods for nonlinear effects and effect modification [36]. Although the choice of spline had little impact, some differences were found for the pooling methods. A one-stage approach using generalized additive mixed effects models (GAMMs) handled splines from differing data ranges and sample sizes better than pointwise meta-analysis or multivariate meta-analysis. However, modeling GAMMs is complex and requires great care.

Belias description of the use of GAMMs is the only guideline on modeling NLEM in a one-stage setting we identified. Some other possible approaches and their challenges have been discussed by Riley and colleagues [2,19].

DeJong describes how nonlinear terms and interactions can be used to model baseline hazard functions and nonproportional hazards in survival analysis [40]. For details on the modeling, they refer to other articles [39,44,45]. Instead of using nonlinear terms the authors suggest achieving proportionality of nonproportional hazards by modeling on a different scale and describe the example of a log-logistic model. If nonlinear terms are used, interpretation can be challenging and the article describes two potentially more clinically meaningful effect measurements, restricted mean survival time difference and percentile ratio. DeJong suggests for one-stage approaches of sufficient sample size, stratification of all parameters is the safest choice and modeling the intervention effect as random to account for heterogeneity.

### 3.3 Sample size calculation for complex relationships in IPDMA

We identified six articles and one book chapter discussing sample size calculation for IPDMAs (Table 3). Three describe simulation-based approaches that allow for modeling of effect modification and specification of nonlinear terms [10,11,46]. Closed form approaches are used in five references [2,11,47–49].

Simmonds first compared the power of three methods to model effect modification: two-stage or one-stage meta-analysis of interaction terms and meta-regression [48]. One-stage models were found to have the largest power but only under a common effects model and ignoring aggregation bias. These are strong assumptions which may not hold. The one-stage approaches presented by Kovalchik and Kontopantelis also do not account for aggregation bias, and can therefore result in too small sample size estimations [46,47].

Riley and colleagues present closed form approaches for continuous and binary outcomes addressing these issues [2,49]. One of the challenges is to estimate the amount of heterogeneity in the size of the interaction in advance and initially the authors suggest assuming an ideal case where no such heterogeneity exists. However, extensions to allow for between-trial heterogeneity are discussed in their book and publication [19,49].

## 4 Discussion

### 4.1 Main findings

In this article we present a review of methodology publications for IPDMA of LEM, nonlinear covariate-outcome relationships, and NLEM including their sample size calculations. Our preceding review of IPD research studies showed that such analyses are common in IPD but rarely implemented correctly or powered for [16]. Easy to follow guidance is needed to support researchers in producing unbiased results that underpin clinical decision making.

We have identified numerous publications describing how to correctly model effect modification at the participant level in a one-or two-stage setting. Many of these have been published in the years considered (2015−2020) although earlier authors (such as Fisher [12]) indicate the challenges in a one-stage setting. It is, therefore, perhaps not surprising that most of the IPDMA research studies published during this time did not implement unbiased procedures although this may be an issue of reporting rather than methodology [16].

Only a few methodology publications on sample size considerations were found and they may not cover all scenarios especially around one-stage approaches and NL. Simulation approaches could be adapted in these cases.

Guidelines on avoiding categorization by analyzing nonlinear covariate-outcome relationships and NLEM are so far focussed on two-stage approaches with some extension for one-stage models.

### 4.2 Limitations

The literature search covered the years 2015 to 2020 and was then updated in November 2022 nonsystematically. It is, therefore, possible that relevant publications during 2021 and 2022 may have been missed. However, we did perform extensive searches through reference lists and citation indices and discussed with experts in the field, thus identifying the most relevant publications.

Additionally, we found little variation in authorship. Most of the articles, including the current review, are authored by a small number of established teams. However, we used a sensitive search strategy, and our exclusion criteria did not discriminate against references by less established authors in the field, for example by favouring high impact journals. We believe this is a comprehensive overview of the currently available methodology guidance.

### 4.3 Best practice recommendations

Based on this review and the preceding review of research studies we make the following recommendations for planning, analysis, and reporting of complex associations in IPDMA.

#### 1 Consider the power for effect modification a priori

Power calculations for assessing effect modification in IPDMA are currently not part of PRISMA-IPD reporting guidelines but help reveal if the IPDMA is worth the time and cost especially if effect modification is part of the main research question. This can be done before IPD collection, based on summary aggregate data from published trials, and under assumptions about true interaction effect sizes [11].

Well defined closed form solutions may not be available for all scenarios, but a simulation-based approach should work in such cases [10]. Easy-to-follow guidance for all scenarios is needed.

#### 2 Choose an appropriate analysis model a priori and consider assumptions and implications

One- and two-stage methods produce similar results if modeling assumptions are matching including how each parameter (treatment effect, covariate effects, intercept, residual variances etc.) is modeled: common, random, or stratified effect. None of the IPDMAs in the preceding review described all these assumptions [16]. However, this choice can strongly impact results and their interpretations [2].

Assessment of effect modification at the participant-level needs to be based on within-trial information alone to avoid the potential for aggregation bias. In cases without any heterogeneity in the estimated effect this is automatically the case. In a two-stage approach this is also automatically done as interaction terms are modeled within each study first and then combined. In the one-stage model within-trial and across-trial information need to be actively separated out, by (1) stratifying all parameters outside the interaction by trial or (2) centering the effect modifier by its trial-specific mean [33].

#### 3 Avoid categorization of continuous covariates

Analyzing continuous covariates instead of categorizing them (1) increases power to detect effect modification if it exists and (2) allows investigation of nonlinearity. If data is shared as continuous then categorization should only be used for exploration but not for primary analysis [10,39].

#### 4 Consider nonlinearity for effect modification of continuous covariates

Nonlinearity in effect modification should be considering when analyzing effect modification by a continuous covariate [2,19,48].

Two main approaches have been suggested using either splines or fractional polynomials. In a single trial setting, little difference has been found between the two methods [50] although they have not been formal compared in an IPD setting. Both approaches are easily modeled in a two-stage IPDMA but challenges arise in a one-stage setting.

#### 5 Adhere to PRISMA-IPD reporting guidelines and include statistical code/formal model specification in publications

When reporting IPDMAs, researchers should adhere to guideline such as PRISMA-IPD and if possible, publish software code or write out the formal model specification to improve understanding and reproducibility especially of one-stage models.

## 5 Conclusion

Guidance on correct IPDMA of complex relationships using one- or two-stage approaches is available and should be used more widely. This will provide higher quality evidence to better support clinical decision making.

## Supplementary Material

Supplementary Material

## Figures and Tables

**Table 1:** Methodological articles focusing on effect modification

Reference	IPDMA approach^a^	Focus^a^	Aggregation bias considered	Recommendation^a^
All outcomes				
Gao 2021 [18]	One- and two-stage	IPDMA of EM in cancer studies	Yes	Prespecify and fully report methods and results of subgroup analyses
Riley 2021 [19]	One- and two-stage	Guideline on analysis of effect modification	Yes	Avoid aggregation bias and categorization of continuous covariates. Presence of effect modification may depend on scale.
Schandelmaier 2020 [4]	One- and two-stage	Credibility of EM analyses	Yes	Tool for judging EM analyses
Riley 2020 [2]	One- and two-stage	Guideline on analysis of effect modification	Yes	Aggregation bias in one-stage analysis can be dealt with by centering or stratification of nuisance parameters
Jiao 2020 [20]	Two-stage	Confidence Distributions based mapping method	Yes	Approach for analyzing multiple related covariates across studies
Belias 2019 [14]	One- and two-stage	Comparison of one- and two-stage models for binary effect modifiers	Yes	Centred one-stage model recommended for binary outcomes
Vo 2019 [21]	Two-stage	Case-mix heterogeneity	Yes	Address case-mix heterogeneity when subgroups are not of interest
Mistry 2018 [22]	One-stage	Tree-based approach, Categorical effect modifiers only	No	Approach for exploring large numbers of effect modifiers, Performs well with large between trial variation
Burke 2017 [23]	One- and two-stage	Differences between one- and two-stage models	Yes	Correctly specified one- and two-stage models perform equally well unless most studies are sparse
Fisher 2017 [13]	One- and two-stage, meta-regression	Validity and reporting of MA of EM	Yes	Meta-analyse interactions, not subgroup effects
Fisher 2015 [24]	Two-stage	Stata command IPDMETAN	Yes	Convenient way to model two-stage IPDMA
Riley 2015 [25]	Two-stage	Multivariate MA of multiple outcomes	Yes	Estimation of within-study correlations in a joint linear regression using Bayesian and frequentist methods
Continuous outcomes				
Papadimitropoulou 2020 [26]	One- and two-stage, meta-regression	Pseudo IPD reconstructed from published aggregate data	Yes	Use of Pseudo IPD is valid if IPD is unavailable and suitable aggregate data about baseline and follow-up is available
da Costa 2019 [27]	One-stage, meta-regression	Methods comparison for MA of subgroup effects	Yes	Allow for the between-trial variation in interaction effects
Noma 2019 [28]	Two-stage	IPDMA of EM for longitudinal data	Yes	Two-stage mixed effects model approach for main and interaction effects
Fokkema 2018 [29]	One-stage	Tree-based approach, Categorical effect modifiers only	No	Approach for exploring large numbers of effect modifiers
Morris 2018 [30]	One- and two-stage	Comparison of one- and two-stage models	Yes	One- and two-stage models perform equally well if correctly specified
Kontopantelis 2018 [31]	One- and two-stage	Comparison of one- and two-stage models	Yes	Use fully specified 1 stage model
Binary outcomes including time-to-event analyses				
Walker 2022 [32]	One- and two-stage	Case study comparison of one- and two-stage models	Yes	Prespecify methods, more real-world explorations are needed
Hua 2017 [33]	One- and two-stage	Addressing aggregation bias	Yes	Separate within-and across-trial variation
Chen 2017 [1]	One- and two-stage	Quantifying heterogeneity	No	Performance of measurements depend on model
Wang 2016 [34]	Two-stage	Visual exploration of continuous effect modifiers. Univariate common effects model only	Yes	Meta-STEPP: Method to identify and model complex EM patterns avoiding categorization.

a IPD, individual participant data; MA, meta-analysis; EM, effect modification.

**Table 2:** Publications on methods for nonlinear covariate-outcome associations and nonlinear effect modification

Reference	Type of outcome	IPDMA approach	Focus^a^	Recommendations^a^
Nonlinear effect modification				
Belias 2022 [36]	Binary	One- and two-stage	4 spline approaches and pointwise MA, multivariate MA, GAMMs	Presence of effect modification may depend on scale. GAMMs are powerful but require careful modeling.
Sauerbrei 2022 [37]	Any	Two-stage	MFPI and pointwise MA (“metaTEF”)	Report analysis using the MethProf-MA profile
Riley 2021 [19]	Any	One- and two-stage	Restricted cubic splines and multivariate MA	Nonlinear treatment-covariate interactions should be investigated. Two-stage multivariate IPDMA of restricted cubic spline functions. Results may depend on the scale.
Riley 2020 [2]	Any	Two-stage	Multivariate MA of splines for NL	Separate within/across trial variation and allow for NL.
Kasenda 2016 [38]	Any	Two-stage	MFPI and pointwise MA	MFPI avoids categorization and allows for nonlinearity in effect modification analyses
Nonlinear covariate-outcome relationships				
White 2019 [39]	Any	Two-stage	FP for nonlinear associations	Modeling nonlinear effects is superior to dichotomization and subgroup analysis
Nonlinear associations in baseline risk				
DeJong 2020 [40]	Time to event	One- and two-stage	Modeling baseline hazard and non-PH	Model non-PH Cox models by rescaling instead of nonlinear or interaction terms.

a MA, meta-analysis; GAMM, generalized additive mixed effects model; MFPI, multivariable fractional polynomial interaction approach; non-PH, nonproportional hazards; NL, nonlinearity; FP, fractional polynomial(s).

**Table 3:** Publications on methods for sample size calculation of LEM, NL or NLEM in IPDMA

Reference	IPDMA approach	Calculation approach	Aggregation bias considered	Recommendation^a^
All outcomes				
Riley 2021 [11]	One- and two-stage	Simulation-based approach, Closed form	Yes	Extension to allow for heterogeneity
Ensor 2018 [10]	Two-stage	Simulation-based approach	Yes	When planning an IPDMA assess power for main effect and effect modification
Kontopantelis 2016 [46]	One-stage	Simulation-based approach	No	Stata command IPDPOWER, but does not separate out within and across-trial relationships, so power will be inflated
Continuous outcomes				
Riley 2020 [2]	Two-stage	Closed form	Yes	Assume no between-study heterogeneity in size of EM
Kovalchik 2012 [47]	One-stage, meta-regression	Closed form	No	Estimate power of IPDMA of effect modification using aggregate data. Power estimates are prone to error due to approximations and amalgamation of within and across-trial information
Simmonds 2007 [48]	One- and two-stage, meta-regression	Closed form	Yes (two-stage), No (one-stage)	Power of each method depends on covariate distribution and sample size, Q statistics measures covariate heterogeneity
Binary outcomes				
Riley 2022 [49]	Two-stage	Closed form	Yes	Improved approximation of variances based on existing aggregate data. Stata and R code are provided
Kovalchik 2012 [47]	One-stage, meta-regression	Closed form	No	Estimate power of IPDMA of effect modification using aggregate data. Power estimates are prone to error due to approximations and amalgamation of within and across-trial information

a EM, effect modification.

## Box 1 Terminology for individual participant data meta-analysis of complex relationships

Here we present some brief explanations of commonly used terms in the literature.

LEM, NL and NLEM:

Interaction: The combined effect of two factors is different than their individual effects. During analysis a multiplicative term is included into the model in addition to the individual factors.

Effect modification: It is a type of interaction between a binary intervention indicator and a covariate called the effect modifier. The effect of an intervention differs depending on the level of the modifier characteristic. During analysis an interaction term between the intervention indicator and covariate is included in the model. If the covariate is categorical, the term is also used when the effect is estimated within subsets of data.

Subgroup effect: The effect of the intervention within a subset of patients usually defined by categorical characteristics. The term subgroup effect is used for analyses including interaction terms or analyses within subsets of data.

Nonlinearity: Estimates are not consistent across varying levels of patient characteristics, either in an effect modification or covariate-outcome relationship.

IPDMA approaches and distributional assumptions:

Two-stage IPDMA: The effect of interest is analyzed in each trial separately and the estimates combined using meta-analysis techniques.

One-stage IPDMA: Data from all trials are analyzed together while accounting for clustering by trial.

Common/Fixed effects: The true effect is assumed to be the same across trials. Differences seen in individual trial estimates are only due to sampling error.

Random effects: The true effects in each trial are assumed to follow a normal distribution allowing for between study variations.

Effects stratified by trial: The effect in each trial is independent from those in other trials.

## Box 2 Data extraction

General: Date of extraction, first author, year of publication, abstract, aims, recommendations, limitations.

Analysis methods: general IPDMA approach (one-/two-stage, common/random/stratified effects), specific methods compared or described.

Approach: Literature review included yes/no, comparison of methods yes/no, methods tested on real datasets yes/no, methods tested using simulation yes/no.

Further supporting references.

## What is new?

### Key findings

- Methodological guidance on individual participant data meta-analysis (IPDMA) of effect modification is available, including how to separate within-trial and across-trial relationships, and how to allow for nonlinearity.
- Further research comparing various proposals for IPDMA of nonlinear covariate outcome relationships or nonlinear effect modification (NLEM) is required.
- Some guidance on a priori sample size requirements is available but not all scenarios are covered.

### What this adds to what is known?

- This review provides an overview of available methodology guidance to address nonlinear covariate-outcome associations (NL) and effect modification in IPDMA.

### What is the implication?

- Comparison of methodological options (e.g., splines, polynomials) for analyzing NL or NLEM is needed.
