# Supplementary material for: Nonlinear effects and effect modification at the participant-level in IPD meta-analysis part 2: methodological guidance is available
Source: J Clin Epidemiol. Author manuscript; Available in PMC 2024 Nov 21. (PMC7616838; doi:10.1016/j.jclinepi.2023.04.014)
Supplement: Supplementary Material [file EMS200039-supplement-Supplementary_Material.docx]

# Appendixexcluded references

| 1. Chen, D. G., et al. (2020). "Relative efficiency of using summary versus individual data in random-effects meta-analysis." Biometrics. |
| --- |
| 1. Hemming, K., et al. (2020). "Extending the I-squared statistic to describe treatment effect heterogeneity in cluster, multi-centre randomized trials and individual patient data meta-analysis." Statistical Methods in Medical Research. |
| 1. Riley, R. D., et al. (2020). "One-stage individual participant data meta-analysis models for continuous and binary outcomes: Comparison of treatment coding options and estimation methods." Statistics in Medicine 39(19): 2536-2555. |
| 1. Belhechmi, S., et al. (2019). "An alternative trial-level measure for evaluating failure-time surrogate endpoints based on prediction error." Contemporary Clinical Trials Communications 15. |
| 1. Fanshawe, T. R. and R. Perera (2019). "Conducting one-stage IPD meta-analysis: Which approach should i choose?" BMJ Evidence-Based Medicine 24(5): 190. |
| 1. Papadimitropoulou, K., et al. (2019). "One-stage random effects meta-analysis using linear mixed models for aggregate continuous outcome data." Research synthesis methods 10(3): 360-375. |
| 1. Schuit, E., et al. (2019). "How often can meta-analyses of individual-level data individualize treatment? A meta-epidemiologic study." International Journal of Epidemiology 48(2): 596-608. |
| 1. Sofeu, C. L., et al. (2019). "One-step validation method for surrogate endpoints using data from multiple randomized cancer clinical trials with failure-time endpoints." Statistics in Medicine. |
| 1. Vo, T., et al. (2019). "Rethinking meta-analysis: Addressing problems of non-transportability when combining treatment effects across patient populations." Revue d'Epidemiologie et de SantePublique 67: S121-S122. |
| 1. Freeman, S. C., et al. (2018). "A framework for identifying treatment-covariate interactions in individual participant data network meta-analysis." Research synthesis methods 9(3): 393-407. |
| 1. Legha, A., et al. (2018). "Individual participant data meta-analysis of continuous outcomes: A comparison of approaches for specifying and estimating one-stage models." Statistics in Medicine 37(29): 4404-4420. |
| 1. Snell, K. I. E., et al. (2018). "Meta-analysis of prediction model performance across multiple studies: Which scale helps ensure between-study normality for the C-statistic and calibration measures?" Statistical Methods in Medical Research 27(11): 3505-3522. |
| 1. Kunkel, D. and E. E. Kaizar (2017). "A comparison of existing methods for multiple imputation in individual participant data meta-analysis." Statistics in Medicine 36(22): 3507-3532. |
| 1. Landau, S., et al. (2017). "Assessing treatment effect moderation in trials of psychological interventions: A case for individual participant data meta-analysis of pooled trials." Trials 18. |
| 1. Thomas, D., et al. (2017). "A comparison of analytic approaches for individual patient data meta-analyses with binary outcomes." BMC medical research methodology 17(1): 28. |
| 1. Egger, M., et al. (2016). "GetReal: from efficacy in clinical trials to relative effectiveness in the real world." Research synthesis methods 7(3): 278-281. |
| 1. Huang, Y. (2016). The ability of aggregate data meta-analysis in predicting individual patient data meta-analysis, ProQuest Information & Learning. 76. |
| 1. Huang, Y., et al. (2016). "Comparing the Overall Result and Interaction in Aggregate Data Meta-Analysis and Individual Patient Data Meta-Analysis." Medicine (United States) 95(14). |
| 1. Kast, J., et al. (2016). "Assessment of covariate effect based on individual patient data vs. Model-based meta-analysis of aggregate data for DPP-4 inhibitors." Clinical Pharmacology and Therapeutics 99: S105. |
| 1. Kaufmann, E., et al. (2016). "Avoiding methodological biases in meta-analysis." Zeitschrift fur Psychologie / Journal of Psychology 224(3): 157-167. |
| 1. Lueza, B., et al. (2016). "Bias and precision of methods for estimating the difference in restricted mean survival time from an individual patient data meta-analysis." BMC medical research methodology 16: 37. |
| 1. Richter, A., et al. (2016). "Simple pooling of data from different studies is increasingly used but not in line with methodological recommendations: A systematic review of methods applied in the field of rheumatoid arthritis." Annals of the Rheumatic Diseases 75: 108. |
| 1. Smith, C. T., et al. (2016). "Individual participant data meta-analyses compared with meta-analyses based on aggregate data." Cochrane Database of Systematic Reviews(9): 56. |
| 1. Song, F. and M. O. Bachmann (2016). "Cumulative subgroup analysis to reduce waste in clinical research for individualised medicine." BMC Medicine 14(1). |
| 1. Waldron, L. and M. Riester (2016). Meta-analysis in gene expression studies. Methods in Molecular Biology, Humana Press Inc. 1418: 161-176. |
| 1. Debray, T. P. A., et al. (2015). "Individual Participant Data (IPD) Meta-analyses of Diagnostic and Prognostic Modeling Studies: Guidance on Their Use." PLoS Medicine 12(10). |
| 1. Debray, T. P., et al. (2015). "Get real in individual participant data (IPD) meta-analysis: a review of the methodology." Research synthesis methods 6(4): 293-309. |
| 1. Riley, R. D., et al. (2015). "Meta-analysis of test accuracy studies: An exploratory method for investigating the impact of missing thresholds." Systematic Reviews 4(1). |
| 1. Riley, R. D., et al. (2015). "Multivariate meta-analysis of prognostic factor studies with multiple cut-points and/or methods of measurement." Statistics in Medicine 34(17): 2481-2496. |
| 1. Riley, R. D., et al. (2015). "Summarising and validating test accuracy results across multiple studies for use in clinical practice." Statistics in Medicine 34(13): 2081-2103. |
| 1. Simmonds, M., et al. (2015). "A decade of individual participant data meta-analyses: A review of current practice." Contemporary Clinical Trials 45: 76-83. |
